# Reframing AI chatbots as cultural and educational catalysts in health professions education

**DOI:** 10.1016/j.jtumed.2025.12.003

**Published:** 2026-01-05

**Authors:** Riza Amalia

**Affiliations:** a Department of Guidance & Counseling, University of Muhammadiyah Sampit, Kotawaringin Timur, Central Kalimantan, Indonesia; b Department of Guidance & Counseling, State University of Malang, Malang, East Java, Indonesia

**Keywords:** AI literacy, Artificial intelligence, Chatbots, Cross-cultural pedagogy, Cultural diversity, Higher education, Longitudinal research, Mixed-methods approach, Physical therapy students

Dear Editor,

The recent cross-national study exploring the use of artificial intelligence (AI)-powered chatbots among physical therapy (PT) students in Egypt, KSA, and Lebanon by El-Sobkey et al. provides valuable insights into how digital technologies are transforming health professions education.^1^ Beyond documenting adoption rates and usage patterns, these findings invite a broader cultural interpretation of how AI integration reflects not only pedagogical innovation but also deeply embedded educational and sociocultural values.

The adoption of AI chatbots in higher education should not be viewed solely as a technological advancement.^2^ Rather, it represents an intersection between technology and culture — a process shaped by collective values, communication norms, and power dynamics within learning environments. In collectivist cultures, such as those prevalent across the Middle East and Asia, students are often socialized to value conformity, respect for authority, and interdependence. Within these contexts, AI chatbots may function as *safe assistants* — non-judgmental digital partners that allow students to seek clarification privately without fear of criticism or loss of face.^3^ This may foster comfort and engagement but can also reinforce passive learning patterns and reduce opportunities for dialogic, critical engagement.

Conversely, in individualistic and low power-distance cultures — commonly observed in Western educational systems — students are typically encouraged to question, debate, and experiment. Here, AI chatbots may serve as *creative collaborators*, enabling learners to test ideas, challenge concepts, and co-construct knowledge in ways that align with critical and self-directed learning paradigms.^4^ These cultural contrasts underscore that AI adoption is not a uniform pedagogical phenomenon but a *context-sensitive educational practice* embedded in different cultural epistemologies of learning and authority.

Language and communication further shape these dynamics. Most AI chatbots, including ChatGPT,^5^ operate predominantly in English, which may inadvertently privilege students with higher linguistic competence and marginalize those who rely on other languages of instruction.^6^ This linguistic bias affects not only accessibility but also the *cognitive framing of inquiry* — how students formulate questions, interpret responses, and construct knowledge through AI-mediated dialogue.^7^ Thus, the relationship between language, culture, and cognition is central to understanding variations in how students trust, depend on, and critically evaluate AI-generated information.

To ensure equitable cognitive skill development, there is a pressing need for cross-cultural frameworks in AI pedagogy.^8^ Such frameworks should consider how cultural orientations toward hierarchy, communication, and uncertainty avoidance influence the ways students interact with AI systems. Educators and policymakers must move beyond the assumption of universal technological benefits and instead design *context-aware AI integration models* that foster both critical thinking and cultural sensitivity. AI literacy training should explicitly address how cultural norms influence patterns of inquiry, ethical reasoning, and intellectual autonomy.

While the present study provides valuable baseline data from Arab countries, its cross-sectional design limits understanding of how AI use evolves across academic trajectories and cultural contexts. Future research should adopt longitudinal and cross-cultural methodologies to capture the dynamic interplay between student development, cultural background, and AI interaction over time. Comparative studies involving diverse regions — including Asia, Africa, Europe, and North America — are essential to identify global and local trends in AI adoption among PT and other health profession students. Such comparisons could illuminate whether patterns observed in collectivist contexts, such as reliance on AI for theoretical learning and limited use in clinical training, are consistent or culturally specific. Moreover, longitudinal approaches can reveal how students’ reliance on AI shifts as they transition from theoretical coursework to practical and clinical stages, where interpersonal skills, empathy, and ethical decision-making become paramount.

To capture these nuances, mixed-methods designs are recommended. Quantitative surveys can map patterns of usage and proficiency, while qualitative approaches — such as ethnographic interviews or digital learning diaries — can uncover the lived experiences and cultural meanings that shape how students engage with AI. This multilevel perspective would help build a more comprehensive understanding of AI's pedagogical impact, accounting for both statistical trends and the sociocultural realities underlying them.
